# Autoregulation Does Not Provide Additional Benefits to a Mixed Session Periodized Resistance Training Program in Trained Men

**DOI:** 10.1519/JSC.0000000000004836

**Published:** 2024-05-30

**Authors:** Sandro Bartolomei, Laterza Francesco, Davide Latini, Jay R. Hoffman

**Affiliations:** 1 Department for Life Quality Studies, University of Bologna, Bologna, Italy; and; 2 Department of Physical Therapy, Ariel University, Ariel, Israel

**Keywords:** strength training, periodization, muscle hypertrophy, power

## Abstract

Bartolomei, S, Francesco, L, Latini, D, and Hoffman, JR. Autoregulation does not provide additional benefits to a mixed session periodized resistance training program in trained men. *J Strength Cond Res* 38(9): 1535–1542, 2024—The aim of this investigation was to study how autoregulation impacted training volume, performance, and muscle size on a 10-week mixed session periodized (MSP) resistance training program, characterized by the inclusion of different training foci in each session. Twenty-four resistance trained men were assigned to an autoregulated mixed session periodized (AMSP group; *n* = 13; age = 26.2 ± 4.9 y; body mass = 82.0 ± 8.7 kg; height = 176.8 ± 6.0 cm) or into an MSP (*n* = 11; age = 24.0 ± 2.6; body mass = 81.3 ± 10.5 kg; height = 174.0 ± 5.4 cm) group. Subjects in both groups trained 5 days per week for 10 weeks and performed the same exercises. The difference between the groups consisted in the use of a perceived recovery-based scale to adjust the individual training volume in the AMSP program. Maximal strength (bench press and squat 1 repetition maximum), power (bench press throw and countermovement jump), and muscle architecture (muscle thickness [MT] of biceps brachii, trapezius, vastus lateralis and vastus medialis) were collected before and after the 10-week training period. In addition, training volume and session load were calculated for each training session. A higher total training volume (*p* < 0.001) was seen in AMSP program compared with MSP program, but no differences (*p* > 0.05) were noted in the average session load. No significant differences between the groups were detected for MT of both upper-body and lower-body muscles (*p's* > 0.05) and lean body mass (*p* = 0.681). No significant differences between the groups were detected for any strength or power measurements. Results of this study indicate that a perceived recovery-based AMSP training program was not more effective than an MSP training program for increasing muscle size and performance in resistance trained men.

## Introduction

Autoregulation is referred to as a purposeful adjustment of training loads in accordance with measurements of the individual's performance or perceived ability to perform (19). The first attempts to adjust the training load based on weekly performance were proposed by DeLorme in 1945 (10). More recently, the use of tests or self-reported scales evaluating the individual's perceived performance capability at the beginning of the training session has been defined as the meta-session autoregulation method (13). This method requires the use of a perceptual based rating scale to adjust the training load for the session (15). Some authors proposed a meta-session autoregulation whereby the individual selects either a hypertrophy or strength- or power-oriented workout depending on their fatigue rating (9,22). This approach has also been defined as nonlinear or flexible daily undulating periodization (22) and was characterized by a specific target in each workout (28). However, these authors reported similar improvements in maximal strength following 9 weeks of flexible DUP resistance training program compared with a regular daily undulating approach (9). A limitation of this approach is the absence of sequencing and phase potentiation, concepts that are fundaments of other training paradigms (e.g., block periodization) (26).

In 2011, a perceived recovery scale developed by Laurent et al. (18) showed a high level of agreement with changes in sprint performance after repeated runs, in trained individuals. This authors (18) reported a high level of agreement between the perceived recovery scale and blood lactate concentration, perceived exertion and session rate of perceived exertion (RPE) following 4 bouts of intermittent sprint. In addition, the level of perceived recovery showed a moderate correlation (*r* = −0.63) with the change in sprint performance (18).

Because recovery represents a crucial factor to optimize adaptations in resistance training (7,16), this strategy may be applied to a flexible periodization approach to adjust training volume within each workout. The rationale is that a reduction in training volume in correspondence of a poor physical recovery may avoid nonfunctional overreaching and chronic overtraining (17). This perceived recovery-based approach differs by other forms of meta-session autoregulation previously used in experimental studies. In this approach, the sequence of the training stimuli within each workout or during the training week are not influenced by the individual perceived recovery status, but the number of sets, and consequently the training volume, is adjusted. Because recovery represents a key factor for training periodization and excessive fatigue may lead to suboptimal adaptations, a perceived recovery-based regulation of training volume may enhance training efficiency.

Recently, mixed session periodized (MSP) resistance training programs have become popular among strength and power athletes, and some authors have supported the effectiveness of this approach for maximal strength and hypertrophy development (5). Because no studies to date have applied a perceived recovery strategy to a resistance exercise program, the aim of this study was to compare the effects of a recovery-based autoregulated mixed session periodized (AMSP) program to a regular MSP program on maximal strength, power, and muscle hypertrophy in resistance trained men. It is hypothesized that a recovery-based approach may optimize adaptations by managing fatigue and reducing suboptimal adaptations during the training period.

## Methods

### Experimental Approach to the Problem

Subjects in this study were assigned to either an AMSP resistance training program or an MSP training group and trained for 10 weeks using the same resistance exercises. The difference between the 2 training programs consisted in the adjustment of the training volume based on the individual perceived recovery status. This strategy was adopted in AMSP group only. Each training program included 5 resistance training sessions per week, and subjects were asked to complete at least 95% of the total number of workouts provided. Subjects were assessed for body composition, muscle architecture, strength, and power performance before (PRE) and at 96 hours following the last training session (POST). They were also asked to record the session rate of perceived exertion (sRPE) following each workout. Based on previous data (3), the estimated sample size was 11 in each group, to detect a between-group difference of 7.2 and 12.1 kg in the 1-repetition maximum (1RM) bench press and squat, respectively, with a power of 0.80. This analysis was performed using the G*POWER 3 software.

### Subjects

Twenty-four experienced resistance-trained men who trained for a minimum of 3 times per week for at least the previous 3 years (mean ± *SD*; 7.0 ± 4.5 years) participated in this study. Inclusion criteria required subjects to bench press at least 1.2 times their body mass (average 1.42 and 1.38 in AMSP and MSP) and squat at least 1.3 times the body mass (1.86 and 1.78 in AMSP and MSP). Twelve of the subjects were strength and power athletes competing in powerlifting (*n* = 3), weightlifting (*n* = 2), and wrestling (*n* = 4) events. Subjects were recruited from university sport science classes and among gym goers. The MSP group included 11 subjects (age = 24.0 ± 2.6 years; body mass = 81.3 ± 10.5 kg; height = 174.0 ± 5.4 cm), and the AMSP group was composed of 13 subjects (age = 26.2 ± 4.9 years; body mass = 82.0 ± 8.7 kg; height = 176.8 ± 6.0 cm). All subjects were between 18 and 35 years and signed an informed consent document after being informed about the potential risks. The study received approval from the Institutional Review Board of the University of Bologna (Protocol n. 0025317 of 2/2/2023).

### Perceived Recovery Scale

Subjects in AMSP were asked to evaluate their recovery status at the beginning of each training session, using a validated perceived recovery scale (18). The subjects were given standardized instructions explaining the use of the perceived recovery scale: a 0–10 representation of the different conditions of individual's recovery. The subjects were asked to draw a line in correspondence of the appropriate number that best described their perceived level of recovery. The evaluation of the perceived recovery status was performed at the beginning of each training session, before starting the warm-up.

### Resistance Training Programs

The 10-week resistance training program for both AMSP and MSP can be observed in Table 1. All subjects exercised 5 days per week. The difference between the training programs consisted in the use of the perceived recovery scale to adjust the training volume within each training session in AMSP. Subjects in MSP were asked to follow the training program without any changes in the number of sets. In both the AMSP and MSP groups, the resistance used for each exercise was selected by the repetitions in reserve (RIR) method. Thus, intensity was selected as the load allowing to perform the requested number of repetitions without reaching volitional failure and to observe the suggested number of RIR (30).

**Table 1:** Training program for the MSP and AMSP groups.*†

Training day: 1	2	3	4	5
Bench press throw (5 sets of 5 reps at 30% 1RM, R: 120 s, MEI)	Box jump (4 sets of 5 jumps, R: 120 s, MEI)	Barbell seal row (4 sets of 5, 50% 1RM, R: 150 s, MEI)	Barbell high pull (4 sets of 5, RIR 5, R:150, MEI)	Box jump (4 sets of 5 jumps, R: 120 s, MEI)
Parallel squat (5 sets of 3, RIR 2, R:120 s)	Bench press (5 sets of 3 reps, RIR 2, R: 150 s)	Barbell seal row (5 sets of 3 reps, RIR 2, R: 150 s)	Inclined bench press (5 sets of 3, RIR 2, R 150 s)	Deadlift (5 sets of 3, RIR 2, R: 150 s)
*Leg extension* (4 sets of 10, RIR1, R: 60 s)	*Dumbbells bench press* (4 sets of 10 reps, RIR 1, R: 90 s)	*Pull-ups* (4 sets of 10 reps. R 90 s)	*Military press* (4 sets of 10, RIR 1, R: 90)	*Deep squat* (4 sets of 10 reps, RIR 1, R: 90 s)
*Leg curl* (4 sets of 10, RIR 1, R 60 s)	*Dumbbell fly* (4 sets of 10 reps, RIR 1, R: 90 s)	*Lat machine* (4 sets of 10 reps, RIR 1, R: 90 s)	*Lateral raise* (4 sets of 10, RIR 1, R: 90 s)	*Leg extension* (4 sets of 10 reps, RIR 1, R: 90 s)
*Standing calf rise* (4 sets of 10, RIR1, R: 60 s)	*Cable triceps extension* (4 sets of 10 reps, RIR 1, R: 60 s)	*Cable pulley row* (4 sets of 10, RIR 1, R: 90 s)	*Front raise* (4 sets of 10, RIR 1, R: 90 s)	*Standing calf raise* (4 sets of 10 reps, RIR 1, R: 60 s)
		*Barbell standing biceps curl* (4 sets of 10, RIR 1, R: 60 s)		

* MEI = maximum explosive intent; R = recovery time; RIR = repetition in reserve.

† High-intensity exercises focused on maximal strength are written in bold, whereas high-volume exercises focused on muscle hypertrophy are written in italics.

**Figure 1.**
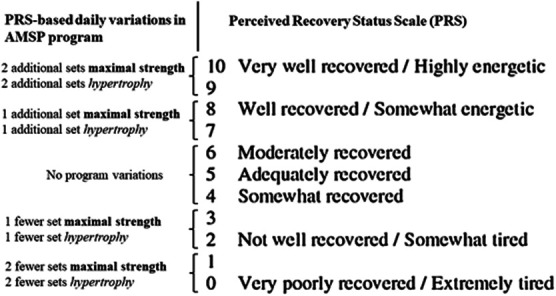
Daily variations in the autoregulated MSP (AMSP) training program based on perceived recovery status scale. (Adapted from Laurent (18).

Both AMSP and MSP training programs consisted of resistance workouts progressing from power exercises performed at a moderate load with maximum explosive intent, to high-intensity exercises, and to high-volume moderate-intensity exercises within the same training session. In AMSP, training volume was adjusted based on the individual's level of perceived recovery status. The number of sets performed for each exercise was adjusted based on the individual perceived recovery score, as reported in Figure 1. Power exercises (jumps, throws) performed at the beginning of each training session were not influenced by perceived recovery scores. Subjects recorded all workouts in a logbook, which was collected by the investigators after each workout. The training sessions were supervised by the study investigators, and a standardized warm-up was performed before each workout and assessment session.

### Strength and Power Testing

Before performance assessments, subjects performed a standardized warm-up consisting of 5 min on a cycle ergometer against a light resistance, 10 body mass squats, 10 body mass walking lunges, 10 dynamic walking hamstring stretches, and 10 dynamic walking quadriceps stretches (2). The 1RM test for the barbell bench press was performed using methods previously described by Bartolomei et al. (4). Briefly, each subject performed 2 warm-up sets using a resistance of approximately 40–60% and 60–80% of his perceived maximum, respectively. For each exercise, 3–4 subsequent trials were performed to determine the 1RM. A 3- to 5-minute rest period was provided between each trial. Trials not meeting the range of motion criteria for each exercise or where technique was not appropriate were discarded. During all other visits, the same standardized warm-up, as described above, was repeated. During each visit, subjects were required to perform a bench press throw test and an isometric bench press test. The bench press throw test was performed using a Smith machine as previously described by Bartolomei et al. (4). Subjects were required to perform the exercise from a supine position with the bar on their chest. They were instructed to push as explosively as possible until complete extension of the arms and to throw the bar as high as possible. Two spotters were placed at each side of the Smith machine to decelerate the bar during descending phase. Subjects pressed loads corresponding to 30% of their 1RM. Two trials were performed with a recovery time of 3 minutes. During all repetitions, an optical encoder (Tendo Unit model V104, Tendo Sports Machines, Trencin, Slovak Republic) measured the mean power expressed by the subjects. Intraclass coefficient for bench press throw was 0.96 (*SEM*: 17.5 w).

The isometric bench press assessment was also performed using a power rack that permitted fixation of the bar. The bench was positioned over a force plate (Kistler 9260, 500 Hz, Winterthur, Switzerland). Subjects were required to position themselves on the bench with their arms at 90° of elbow flexion, and they were not permitted to position their feet on the ground. Elbow angle and grip width were measured to reproduce the same position for all testing sessions. Subjects were asked to press against the bar as hard as possible for 6 seconds. The force expressed against the bar was transmitted by the bench to the force plate, and the peak force was registered. Two isometric bench press attempts were performed with recovery time of 3 minutes between each attempt, and peak force was measured. During all isometric and ballistic measurements, subjects were verbally encouraged by the study investigators. An isometric leg extension assessment was also performed using a custom-built instrumented leg extension machine (3). All leg extension assessments were conducted following the CMJ test. Subjects were secured with adjustable straps to the leg extension machine with hip and knee joint angles at 90° (full extension = 180°). Joint angles were measured using a goniometer while the subject was seated and stabilized to the device, with the right leg attached to the lever arm. A strength gauge (Ergo Tester, Globus Inc., Codogne, Italy) was attached to the end of the lever arm and perpendicular to it. The lever arm was attached to the leg at 15% of tibial length above the medial malleolus. All isometric assessments were performed using the same setting and positioning. Subjects were asked to press against the lever arm as hard as possible for 5 seconds. Each subject performed 2 isometric leg extension attempts, and a recovery time of 2 minutes was provided between each attempt. The peak force generated for each attempt was recorded and used for subsequent analysis. The intraclass correlation coefficients (ICCs) were 0.93 (*SEM*: 89.5 N) and 0.88 (*SEM*: 95.2 N) for isometric leg extension and isometric bench press, respectively.

### Ultrasonography Measurements and Body Composition

Noninvasive skeletal muscle ultrasound images were collected from the subject's right side. Before image collection, all anatomical locations of interest were identified using standardized landmarks for the pectoralis major muscle (Pec), the vastus lateralis muscle (VL), the vastus medialis muscle (VM), the superior part of trapezius muscle (TR), and the bicep brachii muscle (Bic). Pectoralis muscle thickness (PecMT) was measured at the site between the third and fourth costa under the clavicle midpoint (1). The VL MT was measured along its longitudinal distance at 50% from the lateral condyle of the tibia to the most prominent point of the great trochanter of the femur, with the knee bent 10° (6). The landmark for VM was positioned on the muscle belly at 22% of the distance between the upper edge of the patella and the superior iliac spine (8). The landmark for the TR was identified as the midpoint of the muscle belly between T1 and the posterior acromial edge, where the muscle borders were parallel (24). The landmark for the Bic was identified on the anterior surfaces at 60% of the upper arm length (the distance from the acromion process of the scapular to the lateral epicondyle of the humerus) (23). Subjects were asked to lie on the examination table for a minimum of 15 minutes before images were collected. The same investigator performed all landmark measurements for each subject.

A 12 MHz linear probe scanning head (Echo Wave 2, Telemed Ultrasound Medical System, Milan, Italy) was coated with water soluble transmission gel to optimize spatial resolution and used to collect all ultrasound images. The probe was positioned on the surface of the skin without depressing the dermal layer, and the view mode (gain = 50 dB; image depth = 5 cm) was used to take panoramic pictures of the VL. During the measurements, subjects were asked to relax their arm and pectoral muscles and maintain the supine decubitus position. All ultrasound images were taken and analyzed by the same technician. Muscle thickness (MT) measures were obtained using a longitudinal B-mode image. Three consecutive MT images were captured and analyzed for each muscle. For each image, MT was measured with a single perpendicular line from the superficial aponeurosis to the deep aponeurosis. The average of the 3 MT measures was used for statistical analyses. ICC were 0.95 (*SEM* = 0.95 mm), 0.96 (*SEM* = 0.63 mm), 0.96 (*SEM* = 0.93 mm), 0.97 (*SEM* = 0.55), and 0.95 (*SEM* = 0.88) for Pec MT, Trap MT, Bic MT, VL MT, and VM MT, respectively. Anthropometric evaluations were performed before and after the training period. Body measurements included body mass, height, and body fat. Body mass was measured to the nearest 0.1 kg (Seca 769, Seca Scale Corp., Munich, Germany). Body fat percentage was estimated from skinfold caliper (Harpender, CMS Instruments, London, United Kingdom) measures using previously published methods (11).

### Training Volume

Thirty minutes following the conclusion of each training session, subjects responded to the question asked by one of the investigators; “How was your workout?” using a 10-point session RPE (sRPE) scale (12). The scale used the following verbal anchors: 0 = very, very easy, 1 = very easy 2 = easy, 3 = moderate, 4 = somewhat hard, 5–6 = hard, 7–9 = very hard, and 10 = maximal. A session load was calculated for each workout by multiplying the sRPE score by the length of the workout (in mins) (14). Moreover, at the end of the training program, the total perceived training load for both MSP and BP were calculated by summating the session load of each training session performed. The total training volume was also determined for each subject by examining the subjects' training logbooks. Completed training volume was expressed in kilograms.

### Statistical Analysis

A Shapiro-Wilk test was used to test the normal distribution of the data. If the assumption of sphericity was violated, a Greenhouse-Geisser correction was applied. The differences in performance parameters, muscle architecture, and body composition were calculated using a group (AMSP and MSP) × time (PRE − POST) repeated-measures analysis of variance was used to determine interactions and main effects. Pairwise comparisons were performed using the Bonferroni's correction. An independent Student's *t* test was used to compare the total number of repetitions and the average session load between the 2 groups. Where appropriate, percent changes were calculated as follows: [(postexercise mean − preexercise mean)/preexercise mean]. The partial eta-squared statistic was reported as the effect size (ES), and according to Stevens (27), 0.01, 0.06, and 0.14 represent small, medium, and large ES, respectively. Significance was accepted at an alpha level of *p* ≤ 0.05, and all data are reported as mean ± *SD*. All data were analyzed using SPSS20 for Windows (SPSS Inc., Chicago, IL).

## Results

### Strength and Power Testing

**Figure 2.**
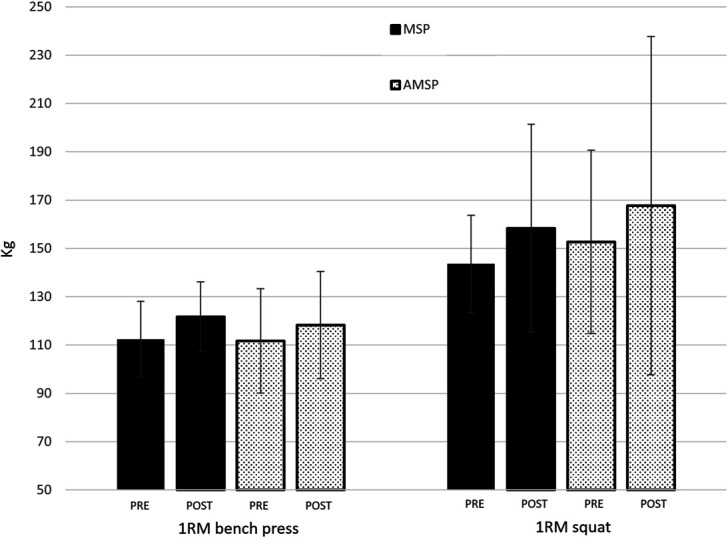
Changes in 1 repetition maximum (1RM) from PRE to POST the training period in MSP and AMSP groups.

**Figure 3.**
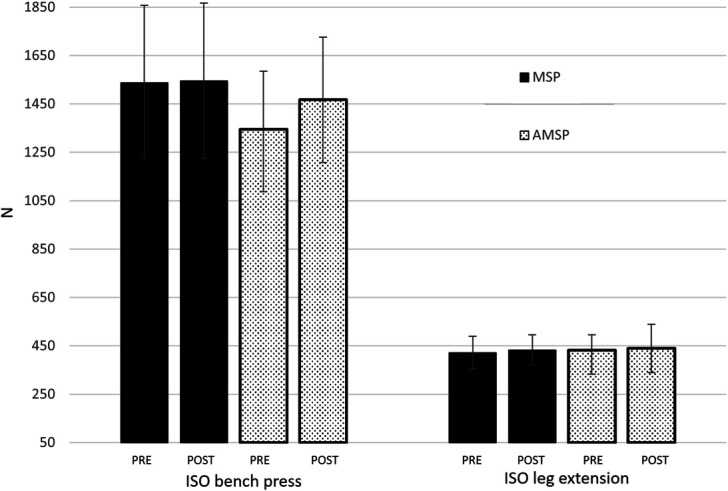
Changes in isometric force from PRE to POST the training period in MSP and AMSP groups. ISO: isometric.

Changes in performance following the AMSP and MSP programs are reported in Figure 2, Figure 3 and Table 2. No group × time interactions were detected for 1RM bench press (*F* = 1.871; *p* = 0.185; *η*^2^ = 0.078), 1RM squat (*F* = 0.377; *p* = 0.546; *η*^2^ = 0.018), isometric bench press (*F* = 3.882; *p* = 0.062; *η*^2^ = 0.172), isometric leg extension (*F* = 0.154; *p* = 0.699; *η*^2^ = 0.008), bench press throw (*F* = 0.396; *p* = 0.536; *η*^2^ = 0.018), and CMJ (*F* = 2.320; *p* = 0.142; *η*^2^ = 0.095). A main effect for the factor time was observed for 1RM bench press (*F* = 66.880; *p* < 0.01; *η*^2^ = 0.752), 1RM squat (*F* = 23.616; *p* < 0.01; *η*^2^ = 0.529), isometric bench press (*F* = 6.925; *p* = 0.015; *η*^2^ = 0.239), and CMJ (*F* = 6.655; *p* = 0.017; *η*^2^ = 0.232). No main effect of time was detected for isometric leg extension (*F* = 0.416; *p* = 0.527; *η*^2^ = 0.021) and bench press throw (*F* = 1.342; *p* = 0.259; *η*^2^ = 0.052).

**Table 2:** Performance parameters evaluated before and after the training period in both mixed session and autoregulated mixed session periodized groups.*

Assessment	Group ►	MSP	AMSP
	Time ▼		
1RM bench press (kg)	PRE	112.4 ± 15.7	111.7 ± 21.6
	POST	121.7 ± 14.4	118.3 ± 22.2
1RM squat (kg)	PRE	143.6 ± 23.3	152.7 ± 41.1
	POST	158.4 ± 20.1	167.7 ± 37.9
Bench press throw (W)	PRE	469.6 ± 43.0	495.2 ± 86.9
	POST	478.7 ± 49.8	497.9 ± 98.0
CMJ (cm)	PRE	41.8 ± 8.1	43.4 ± 6.1
	POST	42.4 ± 7.7	46.0 ± 7.7
ISO bench press (N)	PRE	1,537.5 ± 319.8	1,345.8 ± 238.7
	POST	1,545.5 ± 321.5	1,466.9 ± 259.2
ISO leg extension (N)	PRE	421.4 ± 67.2	432.7 ± 62.2
	POST	432.7 ± 62.2	439.3 ± 99.7

* AMSP = autoregulated mixed session periodized; CMJ = countermovement jump; ISO = isometric; MSP = mixed session periodized; 1RM = 1 repetition maximum.

### Muscle Architecture and Body Composition Measurements

**Figure 4.**
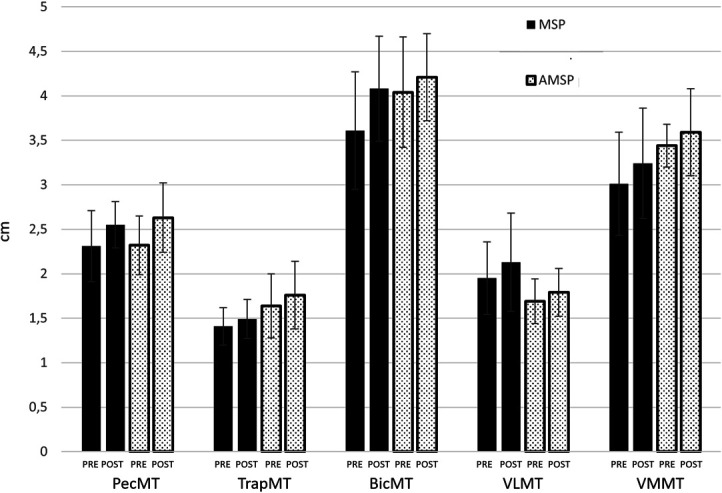
Changes in muscle architecture measures from PRE to POST the training period in MSP and AMSP groups. MT: muscle thickness; Pec: pectoral; Trap: trapezius; Bic: biceps; VL: vastus lateralis; VM: vastus medialis.

Changes in body anthropometric parameters and muscle architecture following both training programs are reported in Table 3. In addition, changes in muscle architecture are depicted in Figure 4. No group × time interactions were identified for PecMT (*F* = 1.127; *p* = 0.300; *η*^2^ = 0.049), TrapMT (*F* = 0.165; *p* = 0.689; *η*^2^ = 0.007), BicMT (*F* = 2.543; *p* = 0.062; *η*^2^ = 0.152), VLMT (*F* = 0.898; *p* = 0.354; *η*^2^ = 0.039), VMMT (*F* = 0.165; *p* = 0.681; *η*^2^ = 0.010), body mass (*F* = 0.960; *p* = 0.338; *η*^2^ = 0.042), fat mass (*F* = 0.758; *p* = 0.393; *η*^2^ = 0.033), and fat-free mass (*F* = 0.175; *p* = 0.681; *η*^2^ = 0.010). A main effect for the factor time was observed for PecMT (*F* = 45.114; *p* < 0.01; *η*^2^ = 0.672), TrapMT (*F* = 4.602; *p* = 0.043; *η*^2^ = 0.173), BicMT (*F* = 20.973; *p* < 0.01; *η*^2^ = 0.488), VLMT (*F* = 10.077; *p* = 0.004; *η*^2^ = 0.314), body mass (*F* = 36.797; *p* < 0.01; *η*^2^ = 0.626), and fat-free mass (*F* = 21.394; *p* < 0.01; *η*^2^ = 0.543). No main effect for the factor time was detected for VMMT (*F* = 4.149; *p* = 0.054; *η*^2^ = 0.159) and fat mass (*F* = 0.224; *p* = 0.549; *η*^2^ = 0.010).

**Table 3:** Anthropometric and muscle architecture measurements before and after the training period in both mixed session and autoregulated mixed session groups.*

Assessment	Group ►	MSP	AMSP
	Time ▼		
Body mass (kg)	PRE	81.3 ± 10.5	82.0 ± 8.7
	POST	83.5 ± 10.2	83.6 ± 9.4
Fat-free mass (kg)	PRE	70.5 ± 5.7	73.1 ± 7.3
	POST	72.4 ± 5.7	75.4 ± 6.9
Fat mass (%)	PRE	12.7 ± 5.1	11.0 ± 2.5
	POST	12.8 ± 5.0	10.7 ± 2.5
PecMT (mm)	PRE	2.31 ± 0.40	2.32 ± 0.33
	POST	2.55 ± 0.26	2.63 ± 0.39
TrapMT (mm)	PRE	1.41 ± 0.21	1.64 ± 0.36
	POST	1.49 ± 0.22	1.76 ± 0.38
BicMT (mm)	PRE	3.61 ± 0.66	4.04 ± 0.62
	POST	4.08 ± 0.59	4.21 ± 0.49
VLMT (mm)	PRE	1.95 ± 0.41	1.69 ± 0.25
	POST	2.13 ± 0.55	1.79 ± 0.27
VMMT (mm)	PRE	3.01 ± 0.58	3.44 ± 0.24
	POST	3.24 ± 0.62	3.59 ± 0.49

* AMSP = autoregulated mixed session periodized; BicMT = biceps brachii muscle thickness; MSP = mixed session periodized; PecMT = pectoral muscle thickness; TrapMT = trapezius muscle thickness.

### Training Volume

The total number of repetitions was significantly higher (*F* = 4.923; *p* < 0.001; *η*^2^ = 0.183) for AMSP (8,519.9 ± 901.2 rip.) compared with MSP (7,893.0 ± 267.8 rip). No significant differences in the average training session load (*F* = 0.875; *p* = 0.360; *η*^2^ = 0.038) were detected between AMSP (508.1 ± 154.3 a.u.) and MSP (445.4 ± 174.3 a.u.).

## Discussion

This study aimed to compare the effects of a MSP resistance training program to an AMSP program on maximal strength, power, and muscle hypertrophy. Subjects in the AMSP group adjusted the training volume based on the perceived recovery score assessed before each training session, whereas subjects in the MSP group were asked to adhere to the program. In both groups, training intensity was prescribed using a repetition in reserve scale. The research hypothesis was that autoregulation may consent a better management of resistance training volume and optimize adaptations.

Results of this study revealed that both MSP and AMSP were effective in improving maximum strength of the upper and the lower body. However, no additional benefits of perceived recovery-based autoregulation of training volume were detected on any strength and power performance assessed. These findings did not confirm the research hypothesis but were in agreement with other authors that investigated autoregulation in the context of a flexible periodization approach (9,26). However, results of this study are not supported by other authors that reported greater improvements in maximal strength following an autoregulating progressive resistance program compared with a program characterized by a linear increase of training intensity (20).

In addition, AMSP was equally effective than MSP for the improvement of MT of upper-body and lower-body muscles and lean body mass. Previous studies have shown that an MSP resistance training program was more effective than a block periodized equated-volume program for maximal strength and muscle hypertrophy gains in trained men (5). However, these results may be influenced by the relative short duration of the training period that may not be enough to obtain delayed and cumulative effects ascribed to block periodized programs (25,29). Conversely, the frequent variation of the training stimuli within each workout that characterizes MSP showed a high effectiveness in stimulating maximal strength and muscle growth, even without planned changes of strategies or a sequence of training phases during a 10-week training period.

In most of the investigations regarding autoregulation in resistance training, subjects were asked to self-regulate the order of the different training sessions within each week (9) or the training intensity (26). The autoregulation used in this program, derived by the Autoregulatory Progressive Resistance Exercise 6RM, developed by Verkhoshansky and Siff (29), and inspired by DeLorme's progressive exercise paradigm (10). However, Autoregulatory Progressive Resistance Exercise and its variants represent within-session autoregulation methods of training intensity (13), whereas the perceived recovery-based AMSP program was developed to adjust the number of sets instead of the training intensity. In this study, training intensity was prescribed using RIR, and loads were selected to prevent exhaustion. Thus, our study was the first to combine a repetition in reserve-based autoregulation of training intensity to a perceived recovery-based autoregulation of training volume (e.g., number of sets). Although the perceived recovery scale represents a valid and ecologic method to assess readiness and expected performance following previous workouts (18), changes in training volume (through variations in the number of sets), have been arbitrarily decided by the investigators at the beginning of the study.

Interestingly, perceived recovery-based autoregulation led to a significant increase in the training volume compared with the regular MSP program, without significant changes in session load. The higher total training volume observed in AMSP (+7.4%) compared with MSP, with nonsignificant changes in the average session load (+12.3% in AMSP), may suggest that autoregulation led to an increase in training volume that was well-tolerated by the subjects. However, this extra volume did not provide for any additional adaptations to the 10-week MSP resistance training.

These results may indicate the subjects tendency to overestimate their physical condition and recovery status from previous training sessions or to underestimate their session RPE. High scores on the perceived recovery scale indeed were associated to increases in training volume compared with the regular MSP program. Perceived recovery is an integrative sensation influenced by physiological, metabolic, and psychological components (17). Thus, because resistance training behavior is strongly associated with exercise motivation (21), perceived recovery scores may be overestimated in resistance training enthusiasts. Further investigations are needed to better understand the influence of motivation on perceived recovery following resistance exercise.

This investigation confirmed the efficacy of the MSP paradigm; however, a perceived recovery-based volume autoregulation MSP program was not able to provide additional adaptations over a 10-week period in trained men. Because this study represents a follow-up of previous investigations focused on MSP training, a limitation of this study consists of the lack of random assignment of the subjects to the 2 study groups. Another possible limitation is represented by the evaluation of the daily individual recovery status, before the beginning of the warm-up. Although significant correlations were detected between pre-warm-up scores of perceived recovery and changes in sprint performance, post-warm-up scores demonstrated stronger correlations with these measurements. However, because perceived recovery scores tend to be more elevated when tested following the warm-up compared with results seen before the warm-up (18), the evaluation of this parameter following the warm-up might result in additional increases in training volume.

**Practical Applications**

The results of this investigation indicated that subjective autoregulation of training volume is effective for enhancing strength, power, and muscle hypertrophy but does not provide additional benefits to an MSP 10-week resistance program with the autoregulation of training intensity. In addition, perceived recovery-based changes in training volume should be accurately selected to avoid nonfunctional increases in total training volume and to optimize performance improvements and muscle growth.
